# PAI-1 is a common driver of aging and diverse diseases

**DOI:** 10.1016/j.bj.2025.100892

**Published:** 2025-07-18

**Authors:** Alireza Khoddam, Toshio Miyata, Douglas Vaughan

**Affiliations:** a Feinberg Cardiovascular and Renal Research Institute, Feinberg School of Medicine Northwestern University, Chicago, USA; b Tohoku University Graduate School of Medicine, Miyagi, Japan

**Keywords:** PAI-1, Aging, Senescence, Cognitive decline, Muscle atrophy

## Abstract

Plasminogen activator inhibitor-1 (PAI-1) is a key driver of aging and contributes to diverse pathologies. This review examines PAI-1's multifaceted contributions to aging. At the cellular level, PAI-1 amplifies senescence, exhausts stem cell niches, and disrupts metabolism. These cellular alterations translate into physiological decline: PAI-1 drives cardiovascular aging by promoting vascular senescence and arterial stiffening, contributes to cognitive decline by impairing amyloid-beta clearance, fuels cancer progression through angiogenesis and immune suppression, and exacerbates muscle atrophy by hindering regeneration. A rare loss-of-function *SERPINE1* mutation extends lifespan, illustrating how lifelong PAI-1 reduction can positively impact the human healthspan. Looking forward, targeting PAI-1 with inhibitors could mitigate senescence, restore stem cell function, improve metabolic profile, enhance physiological health, and promise a longer healthspan.

## 1 Introduction

Plasminogen activator inhibitor-1 (PAI-1), encoded by the *SERPINE1* gene, is a serine protease inhibitor primarily recognized for its role in regulating fibrinolysis by inhibiting tissue-type (tPA) and urokinase-type (uPA) plasminogen activators [1]. Active tPA and uPA facilitate the conversion of plasminogen into plasmin. Subsequently, active plasmin breaks down fibrin, which is an integral meshwork of blood clots. Hence, PAI-1 effectively slows down or prevents clot breakdown. Beyond this hemostatic function, PAI-1 has emerged as a culprit in contributing to aging and age-related diseases [[2], [3], [4]]. A significant body of literature outlines PAI-1's involvement in cellular senescence [5,6], inflammation [7], and tissue remodeling [8,9]. Elevated PAI-1 levels are consistently observed in conditions such as cardiovascular disease [10], metabolic syndrome [11,12], cancer [13], and neurodegeneration [14], suggesting it plays an active role in the aging process.

The association between PAI-1 and aging strengthens over time. Studies across species, including humans [15] and mice [16], demonstrate that circulating PAI-1 level increases progressively with chronological age, paralleling the accumulation of senescent cells and the onset of age-related pathologies [17]. For instance, longitudinal analyses in human cohorts reveal a steep rise in plasma PAI-1 levels after middle age, correlating with increased cardiovascular risk and frailty [3]. This temporal correlation implies PAI-1 may be an active participant in aging, and not merely a passive marker.

Epigenetic aging provides further evidence of PAI-1's involvement. The GrimAge clock, a DNA methylation-based predictor of lifespan and healthspan [18], incorporates *SERPINE1* as the strongest predictor of lifespan with a p-value of 5.4E-28 [19]. Elevated PAI-1 expression is linked to accelerated epigenetic aging, reflecting a feedback loop where cellular stress drives methylation alterations that, in turn, amplify PAI-1 production. This epigenetic signature associates PAI-1 with reduced longevity and heightened disease susceptibility, positioning it as a molecular bridge between genetic regulation and aging phenotypes. Another striking illustration of PAI-1's impact comes from klotho hypomorphic (*kl/kl*) mice, a model of accelerated aging characterized by a roughly 90 % shortened lifespan [20]. These mice exhibit elevated PAI-1 levels in plasma and tissues [21]. Remarkably, genetic deletion or pharmacological inhibition of PAI-1 restores the shortened lifespan of *kl/kl* mice [21]. This rescue highlights PAI-1's potential as a therapeutic target and prompts a deeper investigation into its mechanisms.

This review outlines the cellular and molecular changes such as senescence, stem cell niche disruption, and metabolic dysregulation that underpin physiological alterations across cardiovascular health, cognition, cancer risk, and muscle integrity. Although previous reviews have extensively covered PAI-1 in the context of cardiovascular disease [22], cancer [23], and metabolic dysfunction [11], this review integrates recent evidence with seminal articles in the literature to provide evidence for the model that PAI-1 is not only involved in age-related conditions but is a central driver of the aging process itself. We lastly, present limitations that the field still needs to address. As the global population over 60 is projected to double from 1 billion in 2020 to 2.1 billion by 2050 [24], it may be possible to utilize PAI-1 inhibitors as a unified strategy to target multiple age-related pathological processes through a single target.

## 2 Cellular and molecular alterations

### 2.1 Senescence

**Fig. 1:**
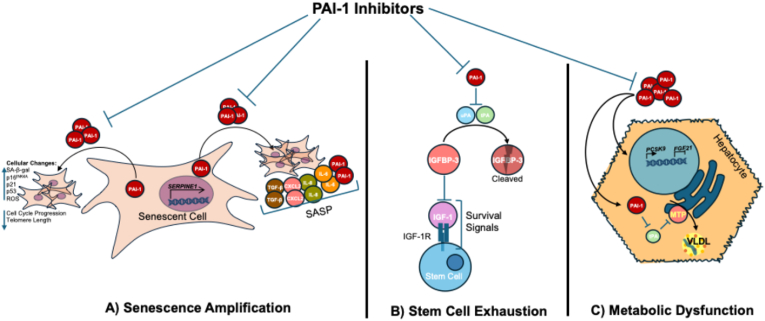
**PAI-1 Drives Aging Pathways via Senescence, Stem Cell Exhaustion, and Metabolic Dysfunction.** (A) Senescence Amplification: PAI-1 promotes p53-driven senescence, SASP (e.g., IL-6, TGF-β), and ROS, causing cell cycle arrest and ECM remodeling. (B) Stem Cell Exhaustion: PAI-1 inhibits uPA/tPA, blocking IGFBP-3 cleavage and IGF-1 signaling, leading to stem cell senescence and niche dysfunction. (C) Metabolic Dysfunction: PAI-1 increases ROS, PCSK9, and FGF21, promoting lipid accumulation (e.g., VLDL) and mitochondrial dysfunction, exacerbating metabolic syndrome.

Cellular senescence, a state of irreversible proliferative arrest induced by stressors like DNA damage, telomere shortening, or oxidative stress, is a hallmark of aging [25]. PAI-1 is intricately tied to this process [Fig. 1A)], serving as both a marker and a driver of senescence across multiple cell types, including fibroblasts, endothelial cells, and epithelial cells [6]. In healthy organisms, cellular senescence facilitates wound healing and halts the replication of aged cells as a mechanism to protect against cancer. PAI-1's expression surges in senescent cells, contributing to the senescence-associated secretory phenotype (SASP), a pro-inflammatory milieu rich in cytokines (e.g., IL-6, IL-8), chemokines (e.g., CCL2), and matrix metalloproteinases (MMPs) that perpetuate tissue dysfunction [26]. Mechanistically, PAI-1 operates downstream of p53, a regulator of senescence. Early studies by Kortlever et al. reveal that PAI-1 overexpression alone can induce senescence in fibroblasts, independent of p53, by inhibiting uPA-mediated proteolysis, which disrupts extracellular matrix (ECM) homeostasis and amplifies cellular stress signals [27]. Additionally, PAI-1 enhances TGF-β signaling, upregulating MMP-2 and MMP-9, which further enforce the senescent state by remodeling the ECM into a rigid, pro-inflammatory scaffold [28]. Although PAI-1 is generally accepted as a SASP component, the exact role of this molecule in inducing senescence remains unsolved. It is not yet clear whether PAI-1 induces senescence through direct interaction with specific membrane receptors, via intracellular signaling mechanisms, or indirectly through secondary molecular pathways. Future studies should focus on deconvolving intracellular and extracellular signaling of PAI-1 to shed light on this mechanism.

The consequences of PAI-1-driven senescence are detrimental, but its reduction offers a counterpoint. Research has shown that interventions targeting senescent cells can extend lifespan of murine models [[29], [30], [31]]. Specifically in *klotho* hypomorphic mice, *Serpine1* haploinsufficiency significantly lowers senescent cell burden, as evidenced by the decreased expression of p16^INK4a^ and p21 in kidney and vascular tissues [21]. This reduction dampens SASP components, mitigating chronic inflammation and fibrosis. Pharmacological approaches complement this genetic finding: TM5441, a selective PAI-1 inhibitor, protects endothelial cells from doxorubicin-induced senescence by suppressing reactive oxygen species (ROS) production and downregulating SASP factors including IL-6 and TNF-α [32]. Furthermore, *in vitro* studies with human fibroblasts show that PAI-1 silencing via siRNA delays replicative senescence, preserving proliferative capacity and reducing β-gal activity, which is an indicator of senescence [33]. These findings suggest PAI-1 not only accelerates senescence but is a target whose inhibition could limit the accumulation of senescent cells, potentially slowing aging-related tissue decline.

### 2.2 Stem cell niches

Stem cell niches are specialized microenvironments that sustain stem cell function, enabling tissue repair and regeneration which are processes that falter with age [34]. As a serine protease inhibitor, PAI-1's inhibition of proteases shapes the ECM landscape of tissues important for stem cell migration. PAI-1's downstream targets of senescence also disrupt these niches through multiple pathways, compromising stem cell vitality and niche integrity [35]. In mesenchymal stromal cells (MSCs), PAI-1 inhibits tPA-dependent cleavage of insulin-like growth factor binding protein-3 (IGFBP-3), a regulator of IGF-1 signaling critical for MSC proliferation and differentiation [Fig. 1B] [36,37]. Ozcan et al. identified this PAI-1-IGFBP-3 axis as a key driver of stress-induced MSC senescence, marked by mitochondrial dysfunction (e.g., reduced ATP production) and upregulation of senescence markers [38]. Additionally, PAI-1 binds vitronectin and engages low-density lipoprotein receptor-related protein 1 (LRP1), altering ECM composition, impairing MSC adhesion, and hindering migration, which are essential for niche maintenance [39,40].

In hematopoietic stem cell (HSC) niches within the bone marrow, PAI-1's effects are equally pronounced [41]. Elevated PAI-1 levels in aged marrow correlate with reduced HSC self-renewal and skewed differentiation toward myeloid lineages, a phenomenon linked to increased ROS and disrupted CXCL12/CXCR4 signaling [42]. Similarly, in muscle satellite cell niches, PAI-1's antifibrinolytic activity impedes ECM degradation, preventing satellite cell activation and proliferation after injury [43]. When PAI-1 levels rise, niches degrade, and regenerative capacity erodes. Conversely, reducing PAI-1 restores niche dynamics as PAI-1 knockout mice exhibit improved HSC function and muscle repair [44,45]. Although current data are limited to select stem cell populations, with potential relevance to other populations such as stem cells, these findings highlight PAI-1 as a regulator of stem cell aging. Modulating its activity may offer a strategy to preserve regenerative capacity and mitigate age-related decline.

### 2.3 Metabolism

PAI-1 also heavily influences metabolism [Fig. 1C]. Excess PAI-1 is linked to the metabolic syndrome, a cluster of conditions such as obesity, insulin resistance, and dyslipidemia that escalate with age [3]. During high fat-induced wight gain, there is experimental evidence suggesting that PAI-1 recruits inflammatory macrophages to visceral white adipose tissue, possibly to clear dead adipocytes [46]. In adipocytes, PAI-1 contributes to inflammation by upregulating pro-inflammatory cytokines (e.g., TNF-α, IL-6), which impair insulin signaling through the PI3K-Akt pathway, reducing GLUT4 translocation to the cell membrane and diminishing glucose uptake [47]. IL-6 has independently been shown to recruit macrophages to adipose tissue and mediate the creation of foam cells [48]. However, no groups have directly demonstrated that PAI-1's role in inflammation is mediated through IL-6's ability to recruit macrophages and is a concept that prompts further investigation. This inflammatory cascade is amplified by PAI-1's inhibition of fibrinolysis, which fosters microvascular dysfunction, limiting oxygen and nutrient delivery to metabolically active tissues, and ultimately exacerbating insulin resistance [10].

PAI-1's metabolic reach extends to systemic effects. In the liver, it enhances hepatic stellate cell activation via TGF-β, promoting fibrosis and impairing lipid metabolism, which contributes to steatosis [49]. Human studies reinforce this: elevated plasma PAI-1 correlates with higher HbA1c in prediabetic patients [50]. More recently, it has been shown PAI-1's target tPA inhibits the ER-embedded Microsomal Triglyceride Transfer Protein (MTP) from synthesizing VLDLs in hepatocytes [51]. Hence, excess PAI-1 sequesters tPA from inhibiting MTP and leads to an increase in VLDL release *in vitro* and in murine models.

Reducing PAI-1 reverses these metabolic impairments. In obese mice, PAI-1 knockout enhances NAD+/NADPH ratio, improving mitochondrial function, glucose tolerance, and reducing adiposity [52]. Pharmacological inhibition of mice with TM5614 mimics this, lowering hepatic *PCSK9* expression while augmenting *FGF21* [53]. Caloric restriction, a proven anti-aging intervention, similarly suppresses PAI-1 [54], implicating a conserved pathway. These findings position PAI-1 as a metabolic switch, where its reduction could mitigate age-related metabolic decline and enhance systemic health.

## 3 Physiological alterations

### 3.1 Cardiovascular aging

**Fig. 2:**
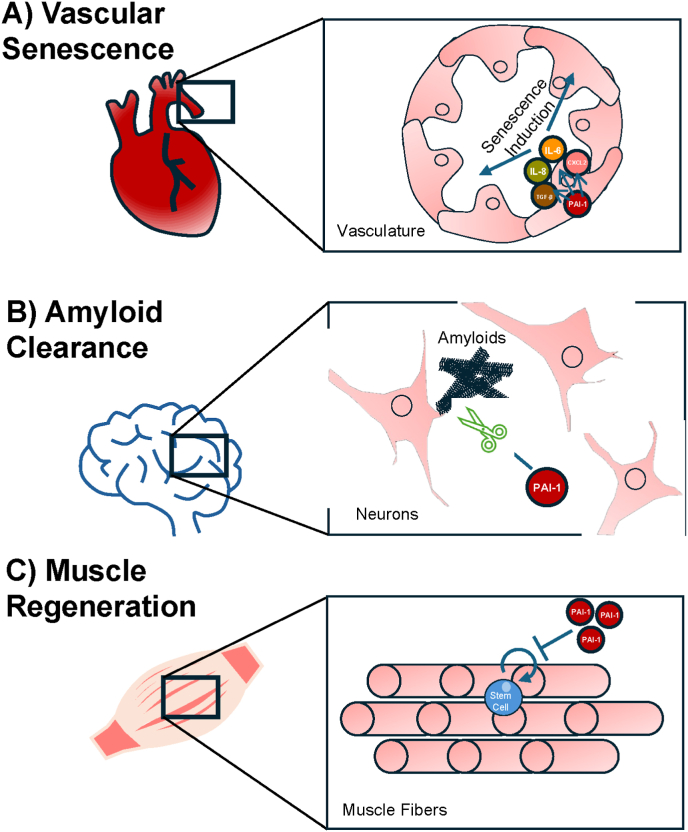
**PAI-1's Impact on Aging-Related Physiological Decline.** (A) Vascular Senescence: PAI-1 promotes endothelial senescence and SASP (e.g., IL-6, IL-8, TNF-α) in the vasculature, driving arterial stiffness and cardiovascular aging. (B) Amyloid Clearance: PAI-1 inhibits tPA, impairing plasmin-mediated amyloid-beta (Aβ) degradation in neurons, contributing to Alzheimer's pathology. (C) Muscle Regeneration: PAI-1 blocks stem cell activation and ECM remodeling in muscle fibers, hindering fiber repair and exacerbating sarcopenia.

Cardiovascular aging characterized by vascular senescence, arterial stiffening, and thrombosis [55] is deeply intertwined with PAI-1 and has been reviewed extensively [22,56] [Fig. 2A]. Briefly, in endothelial cells, PAI-1 contributes to dysfunction by directly binding to endothelial Nitric Oxide Synthase (eNOS), inhibiting the production of nitric oxide (NO) [57]. Since NO is required as a vasodilator maintaining vascular tone [58], the negative interaction of PAI-1 and eNOS promotes vascular stiffness. Additionally, NO is capable of reducing endothelial senescence burden [59]; therefore, PAI-1's inhibition of NO production may mediate further senescence induction. However, data supporting the model that PAI-1 directly inhibits eNOS are limited to *in vitro* systems and thus requires in vivo validation. It is also important to note that since PAI-1's role in a healthy organism is to promote blood clots, a complete loss of PAI-1 is detrimental to the cardiovascular system and causes bleeding disorders [60].

Together with PAI-1 as a component of SASP, accumulation of senescent cells along the vasculature contribute to the arterial pathophysiology seen in advanced age [61]. In vascular smooth muscle cells (VSMCs), it has been suggested that PAI-1 promotes actin filament assembly and contributing to cellular stiffness [62]. In transgenic mice overexpressing a stabilized form of human PAI-1, animals develop age-dependent spontaneous coronary arterial thrombosis [63]. Moreover, treatment with PAI-1 inhibitor TM5441 prevents vascular senescence and perivascular fibrosis in mice treated with l-NAME which inhibits eNOS [64]. Human cohort studies link high plasma PAI-1 to increased cardiovascular disease risk, particularly in older adults [65,66]. These insights frame PAI-1 as a cardiovascular aging architect, with targeted inhibition poised to mitigate its effects.

### 3.2 Cognitive decline

Excess PAI-1 is increasingly implicated in cognitive decline, notably Alzheimer's disease (AD) [67]. In AD, increased PAI-1 plays a critical role in brain cell senescence, particularly in astrocytes, contributing to neurodegenerative pathology [14]. Studies suggest that senescent astrocytes, influenced by elevated PAI-1, may exacerbate neuroinflammation through the release of SASP components like IL-6, which are associated with Alzheimer's disease progression [68]. In humans, aging is the greatest risk factor for late-onset Alzheimer's disease (LOAD), and elevated PAI-1 levels are linked to this condition [69], potentially contributing to cognitive decline. Although specific associations with PAI-1 plasma levels and independence from vascular risk factors require further investigation. Moreover, the stiffening of blood vessels discussed previously is an important physiological driver of cognitive impairment [70].

At the cellular level, PAI-1 inhibits tPA, reducing plasmin generation and hindering Aβ clearance [Fig. 2B] [71,72]. This inhibition could indirectly affect neurodegenerative processes, but its specific role in amyloid-beta clearance or other AD-specific mechanisms requires further investigation. Additionally, PAI-1's contribution to cellular senescence and neuroinflammation in AD, as seen in brain cells, may exacerbate disease progression, though specific links to reactive oxygen species, microglia, or hippocampal damage are not established in the available data. Our current knowledge regarding PAI-1 and its possible role in cognitive function includes experimental studies. In a transgenic mouse with a chimeric human/mouse amyloid precursor protein (APP/PS1 mouse), PAI-1 inhibition improved memory function and reduced the amyloid burden [73]. Other experimental work has begun to suggest that excess PAI-1 is also implicated in cognitive impairments such as PTSD memory formation [74] and late-life depression [75,76]. Interestingly, these studies have also shown that PAI-1 inhibitors are capable of ameliorating these conditions. Collectively, these findings support a model in which PAI-1 acts as an effector molecule in age-related cognitive declines.

### 3.3 Cancer

Aging is an important risk factor for cancer and PAI-1's role in cancer is extensively documented [23,[77], [78], [79]]. Briefly, PAI-1 promotes tumorigenesis by enhancing angiogenesis through VEGF upregulation, protecting tumor cells from apoptosis via PI3K/Akt signaling, and facilitating metastasis by modulating ECM degradation [79]. High PAI-1 expression is a poor prognostic marker in breast [80], lung [81], bladder [82], and endometrial [83,84] cancers. PAI-1's pro-inflammatory effects in the tumor microenvironment, mediated by SASP, further fuel progression. An interesting illustration of this concept is the participation of PAI-1 in the ovarian reproductive system. Within the ovary, PAI-1 tightly regulates ECM remodeling required for folliculogenesis and ovulation [85]. There, dysregulation of PAI-1 remodels the ECM that is permissive to tumor invasion and metastasis [86].

Reducing PAI-1 bolsters anti-tumor immunity. PAI-1 increases PD-L1 expression on cancer cells, allowing for immune evasion and hindering cytotoxic T-cell activity [87]. Interestingly, senescent cells also upregulate PD-L1 to evade immune surveillance [88,89]. This immune evasion mechanism employed by both tumor and senescent cells implies that targeting PAI-1 can address both tumor and dysfunctional senescent cells. It is intriguing to speculate how PAI-1 inhibitors could target multiple aging-related diseases simultaneously. Clinical trials targeting PAI-1 specifically for caner have gained traction, with current trials including chronic myelogenous leukemia [90], metastatic melanoma [91], and non-small cell lung cancer [92], angiosarcoma [93].

### 3.4 Muscle atrophy

Emerging evidence implicates PAI-1 in age-related muscle atrophy known as sarcopenia [Fig. 2C] [94]. Through its canonical role, PAI-1 blocks plasminogen activation and ECM degradation which are important processes essential for muscle repair and regeneration [95]. Indeed, aged muscles are correlated with higher expression of mRNA encoding for PAI-1 [96,97]. Experimental work in murine models have suggested that by promoting plasmin degradation, PAI-1 inhibits the plasmin-mediated activation of Pax7-positive satellite cell proliferation and muscle fiber repair [43]. Perhaps most clinically relevant, pharmacological treatment of old mice with PAI-1 inhibitors demonstrated the ability to prevent muscle atrophy [98]. Although more work is needed to elucidate how PAI-1 hinders muscle growth, current studies highlight PAI-1's role as a sarcopenia mediator and a potential therapeutic target.

## 4 Limitations

Despite substantial progress, critical knowledge gaps remain regarding PAI-1's precise mechanisms of action. Unresolved questions include how excess PAI-1 changes the proteoform landscape of key tissues. As a serine protease inhibitor, PAI-1 can potentially inhibit numerous proteases, and an excess of PAI-1 can create a proteoform landscape that exerts pathological effects. Future studies should include unbiased proteomics and identify downstream targets. Additionally, while PAI-1 inhibitors such as TM5614 demonstrate therapeutic promise in preclinical models, rigorous clinical trials are essential to validate their anti-aging potential as therapeutic agents in humans. Specific guidelines for optimal dosing, treatment duration, and patient selection remain undefined and warrant careful investigation to translate findings into clinical applications. Moreover, it is intriguing to speculate if a combination of PAI-1 inhibitors with other lifestyle changes, such as caloric restriction, can yield synergistic effects.

## 5 Conclusion/outlook

PAI-1 intersects multiple pathways central to aging, including senescence, stem cell exhaustion, metabolic dysfunction, and muscle degradation. Its consistent involvement across these systems highlights it as a key regulator rather than a secondary marker. Remarkably, the field has insight into a natural experiment where we can observe changes associated with a lifelong PAI-1 reduction. A rare loss-of-function *SERPINE1* mutation lowering PAI-1 levels in an Amish population correlates with longer telomeres, enhanced metabolic function, and a 10 % extension of lifespan [99]. With increasing support from genetic, pharmacologic, and preclinical studies, targeting PAI-1 is a realistic therapeutic strategy to decelerate the pace of aging. Inhibitors such as TM5614 have shown potential, and future approaches may include gene editing or epigenetic modulation. These strategies could delay multiple aging-related diseases at once, offering a more unified approach to improving healthspan. By targeting senescence, stem cell function, metabolism, and physiological systems, such strategies could delay cardiovascular, cognitive, oncogenic, and muscular decline. As research advances, PAI-1 emerges not just as an aging culprit but as a gateway to healthier, longer lives.

## Authors’ contributions

All authors contributed substantially to literature review and writing the manuscript.

## Availability of data and materials

Not applicable.

## Financial support and sponsorship

This work was supported by grants from the NIH to D.V. (R35 HL171553) and from the AHA to AK (25PRE1356859).

## References

[1] Loskutoff DJ, Van Mourik JA, Erickson LA, Lawrence D. (1983). Detection of an unusually stable fibrinolytic inhibitor produced by bovine endothelial cells. Proc Natl Acad Sci.

[2] Eren M, Boe A, Klyachko E, Vaughan D. (2014). Role of plasminogen activator inhibitor-1 in senescence and aging. Semin Thromb Hemost.

[3] Cesari M, Pahor M, Incalzi RA. (2010). REVIEW: plasminogen activator inhibitor‐1 (PAI‐1): a key factor linking fibrinolysis and age‐related subclinical and clinical conditions. Cardiovascular Therapeutics.

[4] Schneiderman J, Sawdey MS, Keeton MR, Bordin GM, Bernstein EF, Dilley RB (1992). Increased type 1 plasminogen activator inhibitor gene expression in atherosclerotic human arteries. Proc Natl Acad Sci U S A.

[5] Sun T, Ghosh AK, Eren M, Miyata T, Vaughan DE. (2019). PAI-1 contributes to homocysteine-induced cellular senescence. Cell Signal.

[6] Vaughan DE, Rai R, Khan SS, Eren M, Ghosh AK. (2017). Plasminogen activator inhibitor-1 is a marker and a mediator of senescence. Arterioscler Thromb Vasc Biol.

[7] Bruno MEC, Mukherjee S, Sturgill JL, Cornea V, Yeh P, Hawk GS (2024). PAI-1 as a critical factor in the resolution of sepsis and acute kidney injury in old age. Front Cell Dev Biol.

[8] Declerck P, Gils A. (2013). Three decades of research on plasminogen activator inhibitor-1: a multifaceted serpin. Semin Thromb Hemost.

[9] Sillen M, Declerck PJ. (2021). A narrative review on plasminogen activator inhibitor-1 and its (Patho)Physiological role: to target or not to target?. Int J Mol Sci.

[10] Taeye BD, Smith LH, Vaughan DE. (2005). Plasminogen activator inhibitor-1: a common denominator in obesity, diabetes and cardiovascular disease. Curr Opin Pharmacol.

[11] Altalhi R, Pechlivani N, Ajjan RA. (2021). PAI-1 in diabetes: pathophysiology and role as a therapeutic target. Int J Mol Sci.

[12] Khoukaz HB, Ji Y, Braet DJ, Vadali M, Abdelhamid AA, Emal CD (2020). Drug targeting of plasminogen activator inhibitor-1 inhibits metabolic dysfunction and atherosclerosis in a murine model of metabolic syndrome. Arterioscler Thromb Vasc Biol.

[13] Kortlever RM, Bernards R (2006). Senescence, wound healing, and cancer: the PAI-1 connection. Cell Cycle.

[14] Jiang CS, Rana T, Jin LW, Farr SA., Morley JE, Qin H (2023). Aging, plasminogen activator inhibitor 1, brain cell senescence, and Alzheimer’s disease. Aging Dis.

[15] Yamamoto K, Takeshita K, Kojima T, Takamatsu J, Saito H. (2005). Aging and plasminogen activator inhibitor-1 (PAI-1) regulation: implication in the pathogenesis of thrombotic disorders in the elderly. Cardiovasc Res.

[16] Samad F, Loskutoff DJ. (1996). Tissue distribution and regulation of plasminogen activator inhibitor-1 in obese mice. Mol Med.

[17] Campisi J, d’Adda Di Fagagna F (2007). Cellular senescence: when bad things happen to good cells. Nat Rev Mol Cell Biol.

[18] Horvath S, Raj K (2018). DNA methylation-based biomarkers and the epigenetic clock theory of ageing. Nat Rev Genet.

[19] Lu AT, Quach A, Wilson JG, Reiner AP, Aviv A, Raj K (2019). DNA methylation GrimAge strongly predicts lifespan and healthspan. Aging.

[20] Kuro-o M, Matsumura Y, Aizawa H, Kawaguchi H, Suga T (1997). Utsugi T,et al.Mutation of the mouse klotho gene leads to a syndrome resembling ageing. Nature.

[21] Eren M, Boe AE, Murphy SB, Place AT, Nagpal V, Morales-Nebreda L (2014). PAI-1–regulated extracellular proteolysis governs senescence and survival in *Klotho* mice. Proc Natl Acad Sci U S A.

[22] Sillen M, Declerck PJ. (2020). Targeting PAI-1 in cardiovascular disease: structural insights into PAI-1 functionality and inhibition. Front Cardiovasc Med.

[23] Placencio VR, DeClerck YA. (2015). Plasminogen activator inhibitor-1 in cancer: rationale and insight for future therapeutic testing. Cancer Res.

[24] (2022). Ageing and health.

[25] López-Otín C, Blasco MA, Partridge L, Serrano M, Kroemer G. (2023). Hallmarks of aging: an expanding universe. Cell.

[26] Coppé J-P, Patil CK, Rodier F, Sun Y, Muñoz DP, Goldstein J (2008). Senescence-associated secretory phenotypes reveal cell-nonautonomous functions of oncogenic RAS and the p53 tumor suppressor. PLoS Biol.

[27] Kortlever RM, Higgins PJ, Bernards R. (2006). Plasminogen activator inhibitor-1 is a critical downstream target of p53 in the induction of replicative senescence. Nat Cell Biol.

[28] Samarakoon R, Higgins SP, Higgins CE, Higgins PJ. (2019). The TGF-β1/p53/PAI-1 signaling Axis in vascular senescence: role of caveolin-1. Biomolecules.

[29] Baker DJ, Childs BG, Durik M, Wijers ME, Sieben CJ, Zhong J (2016). Naturally occurring p16Ink4a-positive cells shorten healthy lifespan. Nature.

[30] Xu M, Pirtskhalava T, Farr JN, Weigand BM, Palmer AK, Weivoda MM (2018). et al.Senolytics improve physical function and increase lifespan in old age. Nat Med.

[31] Owens WA, Walaszczyk A, Spyridopoulos I, Dookun E, Richardson GD. (2021). Senescence and senolytics in cardiovascular disease: promise and potential pitfalls. Mech Ageing Dev.

[32] Ghosh AK, Rai R, Park KE, Eren M, Miyata T, Wilsbacher LD (2016). A small molecule inhibitor of PAI-1 protects against doxorubicin-induced cellular senescence. Oncotarget.

[33] Jiang C, Liu G, Luckhardt T, Antony V, Zhou Y, Carter AB (2017). Serpine 1 induces alveolar type II cell senescence through activating p53‐p21‐Rb pathway in fibrotic lung disease. Aging Cell.

[34] Brunet A, Goodell MA, Rando TA. (2023). Ageing and rejuvenation of tissue stem cells and their niches. Nat Rev Mol Cell Biol.

[35] Muñoz-Espín D, Serrano M. (2014). Cellular senescence: from physiology to pathology. Nat Rev Mol Cell Biol.

[36] Elzi DJ, Lai Y, Song M, Hakala K, Weintraub ST, Shiio Y. (2012). Plasminogen activator inhibitor 1—insulin-like growth factor binding protein 3 cascade regulates stress-induced senescence. Proc Natl Acad Sci.

[37] Young K, Eudy E, Bell R, Loberg MA, Stearns T, Sharma D, Velten L, Haas S, Filippi M-D, Trowbridge JJ. (2021). Decline in IGF1 in the bone marrow microenvironment initiates hematopoietic stem cell aging. Cell Stem Cell.

[38] Özcan S, Alessio N, Acar MB, Mert E, Omerli F, Peluso G (2016). Unbiased analysis of senescence associated secretory phenotype (SASP) to identify common components following different genotoxic stresses. Aging.

[39] Czekay R-P, Wilkins-Port CE, Higgins SP, Freytag J, Overstreet JM, Klein RM (2011). PAI-1: an integrator of cell signaling and migration. Int J Cell Biol.

[40] Czekay R-P, Aertgeerts K, Curriden SA, Loskutoff DJ. (2003). Plasminogen activator inhibitor-1 detaches cells from extracellular matrices by inactivating integrins. J Cell Biol.

[41] Harada K, Yahata T, Onizuka M, Ibrahim AA, Kikkawa E, Miyata T (2021). Plasminogen activator inhibitor type-1 is a negative regulator of hematopoietic regeneration in the adipocyte-rich bone marrow microenvironment. Biochem Biophys Res Commun.

[42] Yahata T, Ibrahim AA, Muguruma Y, Eren M, Shaffer AM, Watanabe N (2017). TGF-β–induced intracellular PAI-1 is responsible for retaining hematopoietic stem cells in the niche. Blood.

[43] Bareja A, Lee DE, Ho T, Waitt G, McKay LH, Hannou SA (2024). Liver-derived plasminogen mediates muscle stem cell expansion during caloric restriction through the plasminogen receptor Plg-RKT. Cell Rep.

[44] Krause MP, Moradi J, Nissar AA, Riddell MC, Hawke TJ. (2011). Inhibition of plasminogen activator inhibitor-1 restores skeletal muscle regeneration in untreated type 1 diabetic mice. Diabetes.

[45] Krause MP, Al-Sajee D, D’Souza DM, Rebalka IA, Moradi J, Riddell MC. (2013). Impaired macrophage and satellite cell infiltration occurs in a muscle-specific fashion following injury in diabetic skeletal muscle. PLoS One.

[46] Wang L, Chen L, Liu Z, Liu Y, Luo M, Chen N (2018). PAI-1 exacerbates white adipose tissue dysfunction and metabolic dysregulation in high fat diet-induced obesity. Front Pharmacol.

[47] Cani PD, Amar J, Iglesias MA, Poggi M, Knauf C, Bastelica D (2007). Metabolic endotoxemia initiates obesity and insulin resistance. Diabetes.

[48] Kraakman MJ, Kammoun HL, Allen TL, Deswaerte V, Henstridge DC, Estevez E (2015). Blocking IL-6 trans-signaling prevents high-fat diet-induced adipose tissue macrophage recruitment but does not improve insulin resistance. Cell Metab.

[49] Zheng Z, Nakamura K, Gershbaum S, Wang X, Thomas S, Bessler M (2020). Interacting hepatic PAI-1/tPA gene regulatory pathways influence impaired fibrinolysis severity in obesity. J Clin Investig.

[50] Xu L, Jiang CQ, Lam TH, Bao B, Cheng KK, Thomas GN. (2011). Plasminogen activator inhibitor-1 and HbA1c defined prediabetes: the Guangzhou Biobank Cohort Study-CVD: plasminogen activator inhibitor-1 and HbA1c defined prediabetes. Clin Endocrinol.

[51] Dai W., Zhang H., Lund H., Zhang Z., Castleberry M., Rodriguez M. (2023). Intracellular tPA–PAI-1 interaction determines VLDL assembly in hepatocytes. Science.

[52] Liu S, Li Y, Fan X, Li K, Xu C, Zhang L (2020). Transplantation of adipose tissue lacking PAI-1 improves glucose tolerance and attenuates cardiac metabolic abnormalities in high-fat diet-induced obesity. Adipocyte.

[53] Levine JA, Oleaga C, Eren M, Amaral AP, Shang M, Lux E (2021). Role of PAI-1 in hepatic steatosis and dyslipidemia. Sci Rep.

[54] Sahu U, Villa E, Reczek CR, Zhao Z, O’Hara BP, Torno MD (2024). Pyrimidines maintain mitochondrial pyruvate oxidation to support de novo lipogenesis. Science.

[55] Abdellatif M, Rainer PP, Sedej S, Kroemer G. (2023). Hallmarks of cardiovascular ageing. Nat Rev Cardiol.

[56] Khoddam A., Vaughan D., Wilsbacher L. (2025). Role of plasminogen activator inhibitor-1 (PAI-1) in age-related cardiovascular pathophysiology. J Cardiovascular Aging.

[57] Garcia V, Park EJ, Siragusa M, Frohlich F, Mahfuzul Haque M, Pascale JV (2020). Unbiased proteomics identifies plasminogen activator inhibitor-1 as a negative regulator of endothelial nitric oxide synthase. Proc Natl Acad Sci U S A.

[58] Kolpakov V, Gordon D, Kulik TJ. (1995). Nitric oxide–generating compounds inhibit total protein and collagen synthesis in cultured vascular smooth muscle cells. Circ Res.

[59] Hayashi T, Matsui-Hirai H, Miyazaki-Akita A, Fukatsu A, Funami J, Ding Q-F (2006). Endothelial cellular senescence is inhibited by nitric oxide: implications in atherosclerosis associated with menopause and diabetes. Proc Natl Acad Sci USA.

[60] Fay WP, Shapiro AD, Shih JL, Schleef RR, Ginsburg D. (1992). Complete deficiency of plasminogen-activator inhibitor type 1 due to a frame-shift mutation. N Engl J Med.

[61] Clayton ZS, Rossman MJ, Mahoney SA, Venkatasubramanian R, Maurer GS, Hutton DA (2023). Cellular senescence contributes to large elastic artery stiffening and endothelial dysfunction with aging: amelioration with senolytic treatment. Hypertension.

[62] Khoukaz H.B., Vadali M., Schoenherr A., Ramirez-Perez F.I., Morales-Quinones M., Sun Z. (2024). PAI-1 regulates the cytoskeleton and intrinsic stiffness of vascular smooth muscle cells. Arteriosclerosis Thrombosis Vascular Biol ATVBAHA.

[63] Eren M, Painter CA, Atkinson JB, Declerck PJ, Vaughan DE. (2002). Age-dependent spontaneous coronary arterial thrombosis in transgenic mice that express a stable form of human plasminogen activator inhibitor-1. Circulation.

[64] Boe AE, Eren M, Murphy SB, Kamide CE, Ichimura A, Terry D (2013). Plasminogen activator inhibitor-1 antagonist TM5441 attenuates N ^ω^ -nitro- l -arginine methyl ester–induced hypertension and vascular senescence. Circulation.

[65] Tofler GH, Massaro J, O’Donnell CJ, Wilson PWF, Vasan RS, Sutherland PA (2016). Plasminogen activator inhibitor and the risk of cardiovascular disease: the Framingham Heart Study. Thromb Res.

[66] Heijmans BT, Westendorp RGJ, Knook DL, Kluft C, Slagboom PE. (1999). Angiotensin I–converting enzyme and plasminogen activator inhibitor-1 gene variants: risk of mortality and fatal cardiovascular disease in an elderly population-based cohort. J Am Coll Cardiol.

[67] Barker R, Kehoe PG., Love S. (2012). Activators and inhibitors of the plasminogen system in Alzheimer’s disease. J Cell Mol Med.

[68] Liu R-M. (2022). Aging, cellular senescence, and Alzheimer’s disease. Int J Mol Sci.

[69] Oh J, Lee H-J, Song J-H, Park SI, Kim H. (2014). Plasminogen activator inhibitor-1 as an early potential diagnostic marker for Alzheimer’s disease. Exp Gerontol.

[70] Debès C, Papadakis A, Grönke S, Karalay Ö, Tain LS, Mizi A (2023). Ageing-associated changes in transcriptional elongation influence longevity. Nature.

[71] Angelucci F, Veverova K, Katonová A, Vyhnalek M, Hort J. (2024). Plasminogen activator inhibitor‐1 serum levels in frontotemporal lobar degeneration. J Cell Mol Med.

[72] Liu R-M, Van Groen T, Katre A, Cao D, Kadisha I, Ballinger C (2011). Knockout of plasminogen activator inhibitor 1 gene reduces amyloid beta peptide burden in a mouse model of Alzheimer’s disease. Neurobiol Aging.

[73] Rodriguez G, Eren M, Haupfear I, Viola KL, Cline EN, Miyata T (2023). Pharmacological inhibition of plasminogen activator inhibitor-1 prevents memory deficits and reduces neuropathology in APP/PS1 mice. Psychopharmacology.

[74] Bouarab C, Roullot-Lacarrière V, Vallée M, Le Roux A, Guette C, Mennesson M (2021). PAI-1 protein is a key molecular effector in the transition from normal to PTSD-like fear memory. Mol Psychiatr.

[75] Métivier L, Vivien D, Goy R, Agin V, Bui E, Benbrika S. (2024). Plasminogen activator inhibitor‐1 in the pathophysiology of late life depression. Int J Geriatr Psychiatr.

[76] Jiang H, Li X, Chen S, Lu N, Yue Y, Liang J, Zhang Z, Yuan Y. (2016). Plasminogen activator inhibitor-1 in depression: results from animal and clinical studies. Sci Rep.

[77] Li S, Wei X, He J, Tian X, Yuan S, Sun L. (2018). Plasminogen activator inhibitor-1 in cancer research. Biomed Pharmacother.

[78] Witte JHD, Foekens JA, Brünner N, Heuvel JJTM, Tienoven TV, Look MP (2001). Prognostic impact of urokinase-type plasminogen activator receptor (uPAR) in cytosols and pellet extracts derived from primary breast tumours. Br J Cancer.

[79] Kubala MH, DeClerck YA. (2019). The plasminogen activator inhibitor-1 paradox in cancer: a mechanistic understanding. Cancer Metastasis Rev.

[80] Annecke K, Schmitt M, Euler U, Zerm M, Paepke D, Paepke S (2008).

[81] Bayramoglu A, Gunes HV, Metintas M, Degirmenci I, Guler HI, Ustuner C (2014). Plasminogen activator inhibitor-1 and susceptibility to lung cancer: a population genetics perspective. Genet Test Mol Biomark.

[82] Furuya H, Sasaki Y, Chen R, Peres R, Hokutan K, Murakami K (2022). PAI-1 is a potential transcriptional silencer that supports bladder cancer cell activity. Sci Rep.

[83] Lin L-L, Kost ER, Lin C-L, Valente P, Wang C-M, Kolonin MG (2020). PAI-1-Dependent inactivation of SMAD4-modulated junction and adhesion complex in obese endometrial cancer. Cell Rep.

[84] Lin L-L, Nayak B, Osmulski PA, Wang E, Wang C-P, Valente PT (2024). PAI-1 uncouples integrin-β1 from restrain by membrane-bound β-catenin to promote collagen fibril remodeling in obesity-related neoplasms. Cell Rep.

[85] Devin JK, Johnson JE, Eren M, Gleaves LA, Bradham WS, Bloodworth JR (2007). Transgenic overexpression of plasminogen activator inhibitor-1 promotes the development of polycystic ovarian changes in female mice. J Mol Endocrinol.

[86] Mathews SG, Krishna RB, Murali S, Agarwal P, Rani E, F AM (2024). The role of the plasminogen activator inhibitor 1 (PAI1) in ovarian cancer: mechanisms and therapeutic implications. Global Med Genetics.

[87] Ibrahim AA, Fujimura T, Uno T, Terada T, Hirano K, Hosokawa H (2024). Plasminogen activator inhibitor-1 promotes immune evasion in tumors by facilitating the expression of programmed cell death-ligand 1. Front Immunol.

[88] Wang T-W, Johmura Y, Suzuki N, Omori S, Migita T, Yamaguchi K (2022). Blocking PD-L1–PD-1 improves senescence surveillance and ageing phenotypes. Nature.

[89] Onorati A, Havas AP, Lin B, Rajagopal J, Sen P, Adams PD (2022). Upregulation of PD-L1 in senescence and aging. Mol Cell Biol.

[90] Takahashi N, Kameoka Y, Onizuka M, Onishi Y, Takahashi F, Dan T (2023). Deep molecular response in patients with chronic phase chronic myeloid leukemia treated with the plasminogen activator inhibitor‐1 inhibitor TM5614 combined with a tyrosine kinase inhibitor. Cancer Med.

[91] Fujimura T, Yoshino K, Kato H, Fukushima S, Ishizuki S, Otsuka A (2024). A phase II multicentre study of plasminogen activator inhibitor-1 inhibitor (TM5614) plus nivolumab for treating anti-programmed cell death 1 antibody-refractory malignant melanoma: TM5614-MM trial. Br J Dermatol.

[92] Masuda T, Hirata T, Sakamoto T, Tsubata Y, Ichihara E, Kozuki T (2024). EP.11D.02 an investigator-initiated phase II study of combination treatment of nivolumab and TM5614, A PAI-1 inhibitor for previously treated nsclc. J Thorac Dis.

[93] Fujimura T, Yoshino K, Nakamura M, Kato H, Ito T, Maekawa T (2024). Efficacy and safety of TM5614 in combination with paclitaxel in the treatment of paclitaxel‐resistant cutaneous angiosarcoma: phase II study protocol. Exp Dermatol.

[94] Rahman FA, Krause MP. (2020). PAI-1, the plasminogen system, and skeletal muscle. Int J Mol Sci.

[95] Ahmad K, Shaikh S, Chun HJ, Ali S, Lim JH, Ahmad SS (2023). Extracellular matrix: the critical contributor to skeletal muscle regeneration-a comprehensive review. Inflamm Regen.

[96] Naderi J, Bernreuther C, Grabinski N, Putman CT, Henkel B, Bell G (2009). Plasminogen activator inhibitor type 1 up-regulation is associated with skeletal muscle atrophy and associated fibrosis. Am J Pathol.

[97] Kedlian VR, Wang Y, Liu T, Chen X, Bolt L, Tudor C (2024). Nature Aging.

[98] Aihemaiti A, Yamamoto N, Piao J, Oyaizu T, Ochi H, Sato S (2021). A novel PAI-1 inhibitor prevents ageing-related muscle fiber atrophy. Biochem Biophys Res Commun.

[99] Khan SS, Shah SJ, Klyachko E, Baldridge AS, Eren M, Place AT (2017). A null mutation in *SERPINE1* protects against biological aging in humans. Sci Adv.

